# The Presence of Cholesteryl Ester Transfer Protein (CETP) in Endothelial Cells Generates Vascular Oxidative Stress and Endothelial Dysfunction

**DOI:** 10.3390/biom11010069

**Published:** 2021-01-07

**Authors:** Amarylis C. B. A. Wanschel, Daniele M. Guizoni, Estela Lorza-Gil, Alessandro G. Salerno, Adriene A. Paiva, Gabriel G. Dorighello, Ana Paula Davel, Wayne Balkan, Joshua M. Hare, Helena C. F. Oliveira

**Affiliations:** 1 Department of Structural and Functional Biology, Institute of Biology, State University of Campinas, Campinas 13083-970, Brazil; dani_guiz@hotmail.com (D.M.G.); estelalorza@hotmail.com (E.L.-G.); agsalerno@miami.edu (A.G.S.); adriene.paiva@gmail.com (A.A.P.); gabrieldorighello@gmail.com (G.G.D.); anadavel@unicamp.br (A.P.D.); Ho98@unicamp.br (H.C.F.O.); 2 Interdisciplinary Stem Cell Institute, Miller School of Medicine, University of Miami, Miami, FL 33146, USA; WBalkan@med.miami.edu (W.B.); JHare@med.miami.edu (J.M.H.); 3 Department of Medicine, Miller School of Medicine, University of Miami, Miami, FL 33146, USA

**Keywords:** CETP, oxidative stress, endoplasmic reticulum stress, endothelial dysfunction, superoxide, hydrogen peroxide, mitochondria, Icam-1, Vcam-1, monocytes

## Abstract

Endothelial dysfunction precedes atherosclerosis and is an independent predictor of cardiovascular events. Cholesterol levels and oxidative stress are key contributors to endothelial damage, whereas high levels of plasma high-density lipoproteins (HDL) could prevent it. Cholesteryl ester transfer protein (CETP) is one of the most potent endogenous negative regulators of HDL-cholesterol. However, whether and to what degree CETP expression impacts endothelial function, and the molecular mechanisms underlying the vascular effects of CETP on endothelial cells, have not been addressed. Acetylcholine-induced endothelium-dependent relaxation of aortic rings was impaired in human CETP-expressing transgenic mice, compared to their non-transgenic littermates. However, endothelial nitric oxide synthase (eNOS) activation was enhanced. The generation of superoxide and hydrogen peroxide was increased in aortas from CETP transgenic mice, while silencing CETP in cultured human aortic endothelial cells effectively decreased oxidative stress promoted by all major sources of ROS: mitochondria and NOX2. The endoplasmic reticulum stress markers, known as GADD153, PERK, and ARF6, and unfolded protein response effectors, were also diminished. Silencing CETP reduced endothelial tumor necrosis factor (TNF) α levels, intercellular cell adhesion molecule-1 (ICAM-1), and vascular cell adhesion molecule-1 (VCAM-1) expression, diminishing monocyte adhesion. These results support the notion that CETP expression negatively impacts endothelial cell function, revealing a new mechanism that might contribute to atherosclerosis.

## 1. Introduction

Endothelial dysfunction (ED), which is a central feature of many cardiovascular diseases (CVDs), represents a powerful target in the development of new treatments. During atherosclerosis, ED is directly associated with levels of LDL-cholesterol and inversely correlated with HDL-cholesterol. The successful pharmacologic strategy of reducing LDL-cholesterol through the use of statins has produced a remarkable >20% decrease in CVD events. Treatment with PCSK9 inhibitors reduces LDL-cholesterol by approximately 45–60%, whether used alone or in combination with a statin [1]. However, the risk of a major cardiovascular event in patients with established coronary artery disease remains significant [2,3,4].

Cholesteryl ester transfer protein (CETP) is one of the most potent endogenous regulators of HDL-cholesterol plasma levels. It is a plasma protein that facilitates the transfer of cholesteryl esters from HDL to apoB-containing lipoproteins. In doing so, CETP action leads to a net reduction of HDL-cholesterol in plasma, apparently increasing the risk of atherosclerosis development [5]. HDL levels have cardiovascular protective roles by several means. Besides removing excess cholesterol from the arterial wall, HDL also inhibits lipid oxidation, restores endothelial function, and exerts anti-inflammatory and antiapoptotic actions [6,7]. Therefore, CETP inhibition has been considered a potential target for increasing HDL-cholesterol and reducing CVDs [8,9]. The hope is that the residual CVD risk would be significantly diminished by associating CETP inhibitors with LDL lowering agents, such as statins. However, clinical trials employing at least three different CETP inhibitors (torcetrapib, dalcetrapib, and evacetrapib) have failed to prove this hypothesis and all have been terminated prematurely, either because of increased morbidity/mortality or because of a lack of benefit [10,11,12]. However, the CETP inhibitor anacetrapib showed a highly significant but modest benefit for CVD in the REVEAL trial [13]. Considering that the levels of HDL have several well-established cardiovascular protective roles [14] and that CETP decreases HDL, the present study tested the hypothesis that CETP expression causes endothelial dysfunction and explored possible mechanisms, such as endoplasmic reticulum and oxidative stress.

## 2. Materials and Methods

### 2.1. Human Aortic Endothelial Cell Culture (HAECs)

HAECs, the culture endothelial basal medium (EBM, CC-3121) and endothelial cell growth supplements (EGM, CC-4133) were purchased from Lonza (Walkersville, MD, USA). The cells were grown in EBM containing 15% fetal bovine serum (FBS, R&D, Flowery Branch, GA, USA) at 37 °C in humidified 5% CO_2_, and used for experiments between passages 3 and 11. All siRNAs were purchased from Invitrogen (Eugene, OR, USA). HAECs were transfected with siRNA using Lipofectamine RNAiMAX reagent (Invitrogen), according to the manufacturer’s protocol. Forty-eight hours after transfection, HAECs were harvested for subsequent analyses.

### 2.2. Human Monocytic Cells

Human THP-1 monocytes were purchased from ATCC (Manassas, VA, USA). These cells were cultured in RPMI-1640 medium supplemented with 10% (*v*/*v*) heat-inactivated FBS, 100 U/mL penicillin, 100 μg/mL streptomycin, and 2 mM L-glutamine (all Invitrogen). The culture was maintained at 37 °C in a humidified atmosphere containing 5% CO_2_.

### 2.3. Preparation of Fluorescent THP-1 Cells

To visualize THP-1 cells adhering to the HAECs, fluorescent THP-1 cells were prepared as described by the manufacturer (C2925, Invitrogen). In brief, THP-1 cells were labeled with a fluorescent dye by incubating 1.0 × 10^6^ cells in 10 mL of FBS-free RPMI-1640 and 10 µl of a cell-tracker (final concentration 5 μM) at 37 °C for 45 min. Dye loading was stopped by centrifugation and cells were resuspended in fresh EBM medium.

### 2.4. Adhesion Experiment of THP-1 Cells/Monocyte-EC adhesion

To investigate the CETP effect on the adhesion of THP-1 cells to the endothelial monolayer, HAECs were plated onto Labtek slides (Thermo Scientific, Rochester, NY, USA) and the monolayers transfected with scramble, non-silence (NS) or siCETP. Human THP-1 monocytes previously incubated with 5 μM CellTracker Green (CMFDA) (Invitrogen, Eugene, OR, USA) and resuspended in 500 µl of new EBM medium were placed into each well of a Labtek slide and incubated at 37 °C for 30 min. Next, the THP-1 cell suspension in each well was discarded, and the HAEC monolayer was gently washed three times with 500 μL of phosphate-buffered saline (PBS, pH 7.3) in order to remove non-adherent THP-1 cells. Adherent fluorescence-labeled THP-1 cells were observed under a fluorescence microscope (Nikon, Eclipse Ti, Japan) and quantified.

### 2.5. Animals

Mouse protocols were approved by the University Committee for Ethics in Animal Experimentation (CEUA/Unicamp, protocol # 3507-1) in agreement with the guidelines of the Brazilian College for Animal Experimentation and conformed to the Guidelines on the Handling and Training of Laboratory Animals (published by the Universities Federation for Animal Welfare, U.K., in 1992). Hemizygous human CETP transgenic (Tg) mice (line 5203, C57BL6/J background) [15] expressing a human CETP minigene under the control of its natural flanking sequences were crossed with C57BL6/JUnib mice from the university’s animal care center (CEMIB/UNICAMP) to obtain CETP Transgenic (Tg) and non-transgenic (Ntg) littermates. Male CETP Tg and Ntg, between 12-weeks and 16-weeks of age, were used. Mice were housed in a temperature-controlled room (22 ± 1 °C), on a 12-h light/12-h dark cycle, had free access to water and food and were placed on a standard rodent chow diet (Nuvital CR1, Colombo, Brazil). Mice were genotyped according to the protocol recommended by the Jackson Laboratory (https://www.jax.org/Protocol?stockNumber=003904&protocolID=24829). Terminal experiments were done in mice anaesthetized with 50 mg of ketamine/kg (Parke-Davis, São Paulo, Brazil) and 10 mg of xylazine/kg (Bayer, São Paulo, Brazil).

### 2.6. Vascular Reactivity

Cylindrical segments (rings) of the thoracic aorta (2 mm in length), free of adipose and connective tissue, were mounted in an isolated tissue chamber containing Krebs-Henseleit solution (118 mM NaCl, 4.7 mM KCl, 25 mM NaHCO3, 2.5 mM CaCl2-2H2O, 1.2 mM KH2PO4, 1.2 mM MgSO4-7H2O, 11 glucose, and 0.01 mM EDTA) and gassed with 95% O_2_ and 5% CO_2_. Aortic rings were maintained at a resting tension of 0.5 g at 37 °C, pH 7.4 as previously described [16]. Isometric tension was recorded using an isometric force transducer (MLT0420, ADInstruments, Sydney, Australia) connected to an acquisition system (PowerLab 8/30, ADInstruments). After a 60-min equilibration period, aortic rings were exposed to 125 mM KCl to assess maximal tension. After a washout period, concentration-response curves to the α_1_-adrenoceptor agonist phenylephrine (0.1 nM-10 µM) were obtained and there were no differences. Relaxation concentration–response curves to acetylcholine (1 nM–30 µM) and to the NO-donor sodium nitroprusside (10 pM–3 µM) were generated for the aortic rings contracted with phenylephrine until 50–70% of maximum contraction with 125 mM KCl. Some aortic rings were pre-incubated for 30 min with the nonselective nitric oxide synthase (NOS) inhibitor N-nitro-L-arginine methyl ester (L-NAME, 100 µM, Sigma-Aldrich, St. Louis, MO, USA) before acetylcholine-induced relaxation curves.

### 2.7. Gene-Expression Analysis

Total RNA was extracted from HAECs using the RNeasy mini plus kit, according to the manufacturers’ instructions (Qiagen, Germantown, MD, USA). cDNA synthesis was performed using the high-capacity cDNA reverse-transcription kit, according to the manufacturer’s instructions (Applied Biosystems). Quantitative PCR was performed using Taqman Universal Master mix in an iQ5 real-time PCR (qRT-PCR) detection system (Bio Rad). All samples were run in triplicates and normalized to a GAPDH/18s endogenous control. Relative fold-change was calculated using the ΔΔCt method. Taqman Gene-expression assays were performed for the following genes: CETP (Hs00163942_m1), TNFA (Hs00174128_m1), CD106 (Hs01003372_m1), CD54 (Hs00164932_m1), and ATF6 (Hs00232586_m1).

### 2.8. Subcellular Fractionation and Western Blot Analysis

Subcellular fractionation was performed using the Cell Compartment Kit (Qiagen, 37502), according to the manufacturer’s instructions. Whole lysates of HAEC were prepared by scraping cells with lysis buffer containing protease inhibitors (Qiagen). Protein concentration was determined by the bicinchoninic acid (BCA) method. Proteins were separated on 8% or 15% gels by SDS polyacrylamide gel-electrophoresis (PAGE) and transferred onto Immobilon-P membranes (Millipore, St. Louis, MO, USA). Primary antibodies used were anti-p47 (Invitrogen, PA5-27821), NOX2 (Invitrogen, MA5-18052), and L-type Ca^2+^ channels (sc-25686). Bound primary antibodies were detected with horseradish-peroxidase-conjugated secondary antibodies (Sigma), which was followed by enhanced chemiluminescence (Thermo-Scientific).

The HAECs or aortic tissues were homogenized in RIPA lysis buffer containing a 1% protease inhibitor cocktail. Samples were analyzed by electrophoresis on a 10% sodium dodecyl sulphate containing polyacrylamide gel and then transferred to nitrocellulose/polyvinylidene difluoride (PVDF) membrane. For plasma membrane protein analyses, cells were lysed using Qproteome Plasma Membrane Protein Kit (37601, Qiagen, Germantown, MD, USA). Western-blot analyses were carried out according to standard protocols with primary antibodies raised against CETP (10240, Cayman Chem. Co., Ann Arbor, MI, USA), VCAM-1 (V9263, Sigma), ICAM-1 (HPA002126, Sigma), eNOS (ABS229, Millipore, St. Louis, MO, USA), p-eNOS serine1177 (612392, BD, Franklin Lakes, NJ), SOD2 (ab13533-50, Abcam, Cambridge, MA, USA), AKT1 (sc-8312, Santa Cruz), p-AKT1 serine 473 (sc-7985, Santa Cruz), ERK1 (sc-94, Santa Cruz, Dallas, TX), p-ERK (sc-7383), Caveolin-1 (sc-5310, Santa Cruz), Caveolin-3 (sc-5310, Santa Cruz), nNOS (sc-648, Santa Cruz), p47 (Invitrogen, PA5-27821), NOX2 (Invitrogen, MA5-18052) L-type Ca2^+^ channel (sc-25686), PERK (Cell Signaling, 3192), p-IRE (NB100-2323, Novus, Centennial, CO), IRE (14C10, Cell Signaling). Beta-actin (AM4302; Thermo Scientific, Waltham, MA), α-tubulin (TU-02, Santa Cruz Biotech.), HSP90 (4874, Cell Signaling, Danvers, MS) and GAPDH (sc-25778, Santa Cruz) were used as internal controls depending on the tissue/condition. Immunodetection was performed using an enhanced chemiluminescence detection kit (Thermo Scientific). Band intensities were quantified using ImageJ software (National Institute of Health, Bethesda, MD, USA).

### 2.9. Measurements of Superoxide in HAECs

The fluorescent dye dihydroethidine (DHE, Molecular Probes) was used to evaluate superoxide generation, as previously described [12,13]. DHE exhibits blue-fluorescence in the cytosol until oxidized, where it intercalates within the cell’s DNA, staining its nucleus a bright fluorescent red. HAECs were incubated at room temperature for 30 min with DHE (3 μM). After washing with PBS, cells were fixed with 2% paraformaldehyde for 5 min at 4 °C. Samples were then mounted in Prolong Gold anti-fade (Invitrogen). Images were obtained using a Zeiss LSM-710 confocal microscope. Nuclear fluorescence captured at 562 nm was quantified using the Image-Pro plus software (MediaCybernetics, Silver Spring, MD, USA) and normalized to a cytosolic fluorescence.

### 2.10. Measurements of Superoxide in Aortas

DHE was used to evaluate in situ superoxide generation, as previously described [16,17]. Nuclear fluorescence captured at 562 nm was quantified using the Image-Pro plus software (MediaCybernetics, Silver Spring, MD, USA) and normalized to cytosolic fluorescence. Aortas were embedded in a freezing medium and transverse sections (10 µm) of frozen arteries were obtained using a cryostat (Company), collected on glass slides, and equilibrated for 10 min in Hanks solution (1.6 mM CaCl_2_, 1.0 mM MgSO_4_, 145.0 mM NaCl, 5.0 mM KCl, 0.5 mM NaH_2_PO_4_, 10.0 mM dextrose, 10.0 mM HEPES, pH 7.4) at 37 °C. Fresh Hanks solution containing DHE (2 µM) was topically applied to each tissue section and the slices were incubated in a light-protected humidified chamber at 37 °C for 30 min. Negative control sections received the same volume of Hanks solution in the absence of DHE. Images were obtained with a Leica DMI600B microscope. The number of nuclei labeled with 4,6-diamidino-2-phenylindole (Dapi, positive nuclei) along the vascular wall was automatically counted using Image J software.

### 2.11. Production of H_2_O_2_ by HAECs

To assay the production of H_2_O_2_, 0.5 × 10^5^ peritoneal macrophage cells were incubated in 96-well cell culture plates. On the day of the experiment, cells were incubated in a mixture contained 50 μM Amplex Red reagent (Invitrogen, Waltham, MA, USA) and 0.1 U/mL horseradish peroxidase in Krebs-Ringer phosphate (145 mM NaCl, 5.7 mM sodium phosphate, 4.86 mM KCl, 0.54 mM CaCl_2_, 1.22 mM MgSO_4_, 5.5 mM glucose, pH 7.35). This assay was conducted in the presence and absence of catalase (1000 U/mL). Fluorescence was monitored over time with a temperature-controlled SpectraMax M3 Microplate Reader (Molecular Devices LLC, San Jose, CA, USA) using excitation and emission wavelengths of 560 and 590 nm, respectively. Results were expressed as μM of H_2_O_2_/60 min/0.5 × 10^5^ peritoneal macrophage cells using the standard curve established for each assay with known molar concentrations of H_2_O_2_.

### 2.12. Production of H_2_O_2_ by Aortas

Aorta segments were incubated in 96-well cell culture plates with a mixture contained of a 50-µM Amplex Red reagent (Invitrogen) and 0.1 U/mL HRP in Krebs-Ringer phosphate (145 mM NaCl, 5.7 mM sodium phosphate, 4.86 mM KCl, 0.54 mM CaCl_2_, 1.22 mM MgSO_4_, 5.5 mM glucose, pH 7.35). Fluorescence was monitored over time with a temperature-controlled SpectraMax M3 Microplate Reader (Molecular Devices LLC) using excitation and emission wavelengths of 560 and 590 nm, respectively.

### 2.13. Reduced (GSH) and Oxidized (GSSG) Glutathione Levels

Aorta (50 mg) of GSH and GSSG were assayed separately according to the fluorometric ortho-phthalaldehyde (OPT) method [18]. This method is based on the principle that OPT reacts with GSH and GSSG, at pH 8.0 and pH 12, respectively, to yield a highly fluorescent product, which can be activated at 350 nm with an emission peak at 420 nm. GSSG levels were determined after sample treatment with N-ethylmaleimide to remove GSH completely. The concentrations of GSH and GSSG in samples were calculated according to standard curves prepared with GSH and GSSG, respectively.

### 2.14. Immunofluorescence Staining

For CETP localization experiments, HAECs were plated on Labtek slides and transfected with siRNA. Forty-eight hours after transfection, HAECs were washed with PBS and then fixed with 4% paraformaldehyde (PFA) for 10 min, blocked with 10% bovine serum albumin in PBS for 1 h, and stained with monoclonal antibody against CETP (Sigma, C7365), Ant1/2 (Abcam, clone AB.H10, # ab18180), p47 (Invitrogen, PA5-27821), NOX2 (Invitrogen, MA5-18052), Grp78/BIP (Abcam, ab-21685), GADD153 (Santa-Cruz Biotechnology, sc-793), overnight. After three washes with 1 × PBS, cells were incubated with Alexa Fluor secondary antibody A11011 and A11029 (Alexa Fluor-Invitrogen) for 1 h at RT. For membrane and cytoplasm co-localisation studies, cells were subsequently stained with Alexa Fluor 555 Wheat Germ Agglutinin (W32464, Life Technologies) or phalloidin conjugated to Alexa Fluor 568 (A12380, Invitrogen), for 1 h or 30 min, respectively, at RT. To perform membrane cholesterol and lipid rafts detection we used filipin (Abcam, 133116) and CTB (Sigma, C1655) staining, respectively. Cells were fixed in 1% PFA for 30 min on ice. Labteks were mounted on glass slides with mounting media containing DAPI (# H-1200, Vectashield, Vector Laboratories, San Jose, CA) or ProLong (P36980, Invitrogen). All images were analyzed using Leica DMI600B and Leica Upright LSM780-NLO microscopes. High magnification z-stack images were recorded for studying cell morphology changes. All gains for the acquisition of comparable images were maintained at a constant level. Image analyses were performed using ImageJ (NIH) and Adobe Photoshop CS5.

### 2.15. Measurement of Mitochondrial Superoxide Production in HAECs

HAECs were allowed to grow until 70% confluence and transfected with siCETP on 8-well Labek slides. Cells were pre-incubated with 5 μM of the mitochondrial superoxide indicator MitoSOX (Molecular Probes, Invitrogen) at 37 °C for 30 min. For live cell imaging, cells were then rinsed in warm culture medium and stained with Mitotracker Green (Molecular Probes, Invitrogen) (100 nM) at 37 °C for 15 min, which was followed by three washes with warm medium. Nuclei were counterstained with DAPI for 10 min. Cells were mounted onto glass slides with Mounting Medium Vectashield (Vector Laboratories). Images of cells were acquired using a Leica LSM 780 confocal microscope with 63X objective, which was followed by measurement of the average cell area of at least 10 cells per field using ImageJ software.

### 2.16. Flow Cytometer Analysis

Reactive oxygen species (ROS) production was determined by CellRox, Deep Red oxidative stress reagent (C10491, Life Technologies, Carlsbad, CA, USA). After transfection, cells were cultured in the medium for 48 h and then collected and treated with diphenyleneiodonium chloride (DPI, 10 µM, Sigma Aldrich, St. Louis, MO, USA), or 2-(2,2,6,6-Tetramethylpiperidin-1-oxyl-4-ylamino)-2-oxoethyl)triphenylphosphonium chloride (mito-TEMPO, 10 μM, Sigma Aldrich) or Apocynin (100 μM) for 2 h. Staining was performed by incubating cells at 37 °C with 5 μM CellRox following the manufacturer’s protocols (Invitrogen) and directly analysed without fixing. Cell analysis was performed in FACS Canto-II (BD Biosciences). For mean fluorescence intensity (MFI) determination, we used FlowJo flow cytometry analysis software. MFI refers to the fluorescence intensity of each event (on average) of the selected cell population in the chosen fluorescence channel.

### 2.17. Statistical Analysis

Data are presented as a mean ± standard error of the mean (SEM) (n is provided in the figure legends) and the statistical differences were evaluated by Student’s t-test versus the control group. Dose-response curves for vascular reactivity assays were analyzed by two-way ANOVA. Results were considered significantly different at *p* < 0.05.

## 3. Results

### 3.1. CETP Impairs the Endothelium-Dependent Relaxation

**Figure 1:**
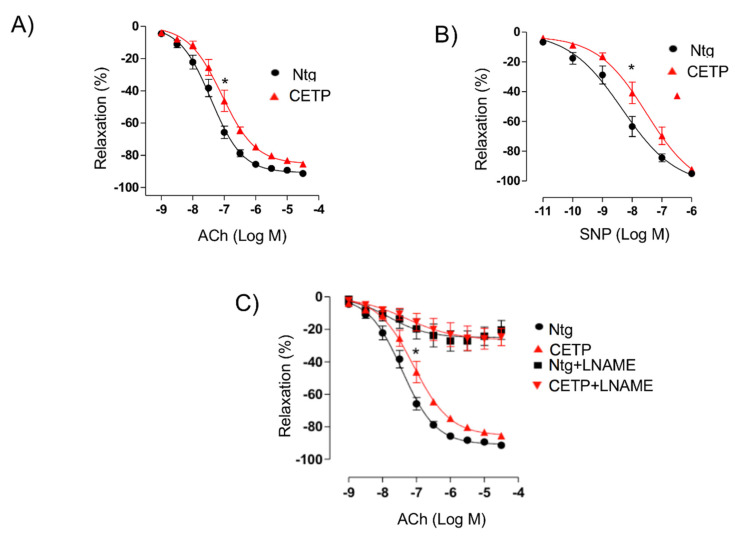
Cholesteryl ester transfer protein (CETP) expression impairs endothelium-dependent relaxation. Endothelium-dependent relaxation in response to acetylcholine (Ach, panel (**A**)) or to NO donor sodium nitroprusside (SNP, panel (**B**)) in isolated aortic rings pre-contracted with phenylephrine. Acetylcholine-induced vessel-relaxation is dependent on nitric oxide synthase (eNOS) activity, since it is abolished in the presence of N(ω)-nitro-L-arginine methyl ester (L-NAME, panel (**C**)) in aortas of both CETP-transgenic (CETP) and non-transgenic mice (Ntg). Data are mean ± SEM (*n* = 10 per group). * *p* < 0.05 for pD2 and Emax by two-way ANOVA.

On aortic rings pre-contracted with 10^−5^ M phenylephrine, the maximal effect (Emax) and the half maximal effective concentration (pD_2_) of acetylcholine-induced relaxation were both impaired in CETP Tg compared to Ntg mice (Emax: Ntg = 91.3 ± 1.8 vs. CETP = 85.5 ± 1.4%, *p* < 0.05, pD2: Ntg = −7.4 ± 0.1 vs. CETP= −7.1 ± 0.1, *p* < 0.05) (Figure 1A). The response of aortas to sodium nitroprusside (SNP) was also impaired in CETP Tg mice (Emax: Ntg = 95.1 ± 0.7 vs. CETP = 92.1 ± 1.4%, *p* < 0.05, pD2: Ntg= −8.4 ± 0.2 vs. −7.7 ± 0.2, *p* < 0.05) (Figure 1B). Pre-treatment with the non-selective NOS inhibitor (L-NAME) blocked acetylcholine-induced relaxation in both groups by abolishing the difference between groups (Figure 1C). Therefore, aortas from CETP Tg mice showed decreased responsiveness and sensitivity to both SNP and acetylcholine, as demonstrated by the significant rightward shift of the concentration-response curves compared with aortas from Ntg mice.

### 3.2. Effects of CETP on Caveolin/NOS Association, eNOS Activation

**Figure 2:**
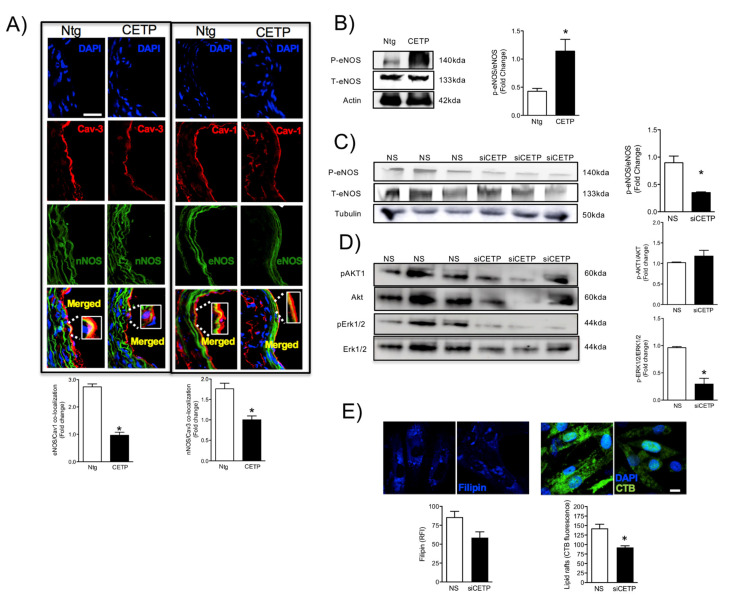
CETP reduces Caveolin/NOS association and increases eNOS phosphorylation. Representative images of mouse aortas (**A**) showing the effects of CETP on NOS/Caveolin interactions, and quantification is shown below. Results are Mean ± SEM, *n* = 4–5 * *p* <0.05 (scale bar = 50 µm). (**B**) Western-blot analysis of phosphorylated (Ser1117) and total eNOS in aortas from CETP transgenic and non-transgenic (Ntg) mice or (**C**) HAECs transfected with siCETP or control scrambled siRNA. (**D**) Phosphorylated and total AKT1 and ERK1/2 in HAECs. Results are mean ± SEM, *n* = 3. * *p* <0.05. (**E**) HAECs transfected with siCETP or control scrambled siRNA were stained with filipin or fluorescent-labeled CTB to detect levels of surface membrane lipids. Quantification of filipin or CTB binding efficiency (*n* = 10, 2 experiments, * *p* < 0.001), scale bar = 10 µm. HAECs, human aortic endothelial cells. CETP, cholesteryl ester transfer protein. NS, non-silence. siCETP, silenced CETP. CTB, cholera toxin subunit B.

Next, we examined the effects of CETP on the NOS/Caveolin co-localization in aortas by immunohistochemistry. NOS has evolved to be tightly controlled by co-translational and post-translational modifications, phosphorylation on multiple residues, and regulation by protein-protein interactions. Endothelial (eNOS) and neuronal (nNOS) NOS can interact with various proteins in their ‘less active’ and ‘more active’ states. eNOS associates with the caveolae coat protein caveolin-1 (Cav-1) and nNOS with caveolin-3 (Cav-3). Here, we observed that CETP expression reduced both aortic eNOS/Cav-1, and nNOS/Cav-3 association (Figure 2A). In addition, CETP expression increased eNOS phosphorylation in aortas (Figure 2B). In HAECs, eNOS phosphorylation was reduced by 60% under CETP silencing (Figure 2C). The mechanism underlying phosphorylation of eNOS appears to be via the ERK signaling pathway as indicated by reduced Erk1-2 phosphorylation, while no changes in AKT phosphorylation levels after CETP inhibition were detected (Figure 2D). Interestingly, glycosphingolipid, which is a typical component of caveolae, measured by cholera toxin subunit B (CTB) binding, was downregulated after CETP silencing in HAECs (Figure 2E). In addition, a tendency to decreased membrane cholesterol content stained by filipin was observed (Figure 2E). These results suggest that CETP impact on the composition and amount of surface membrane lipid microdomains, such as lipid rafts (ex.: caveolae), this may be an additional mechanism of regulation of NOS activation.

### 3.3. CETP is Involved with Increased Vascular ROS

**Figure 3:**
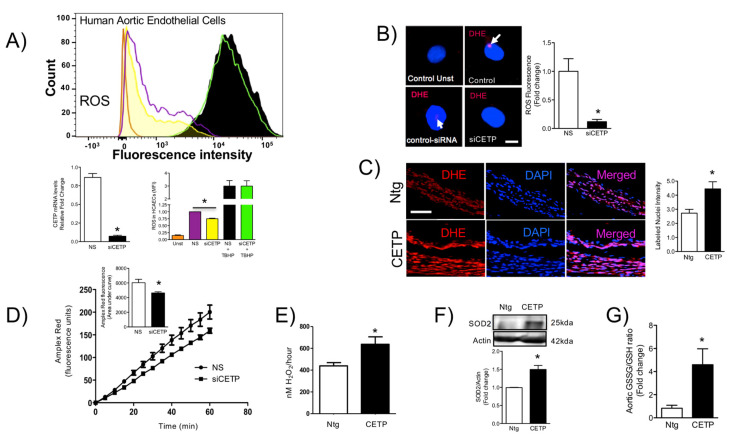
CETP expression induces oxidative stress in vivo and in vitro. (**A**) mRNA levels of CETP in HAECs transfected with CETP or control scrambled siRNA (* *p* < 0.0001). In order to measure ROS production, HAECs were transfected with CETP, or control scrambled siRNA and, immediately after, stimulated with TBHP as a positive control. HAECs were then incubated with cellROX and expression of ROS was measured via fluorescent activated cell sorting (FACs). HAECs transfected with siCETP lowered ROS production by 25%. Data are mean ± SEM, three experiments each in duplicate or triplicate, * *p* < 0.01 by an unpaired Student’s T test. (**B**,**C**) Superoxide production was determined in (**B**) HAECs (*n* = 10, 2 experiments, * *p* < 0.01, scale bar = 10 µm) and (**C**) mouse aortas (*n* = 3–4, * *p* < 0.05, scale bar = 50 µm) using dihydroethidine (DHE) probe. The fluorescence signal was evaluated as the intensity of fluorescence per pixel in at least 10 cells or normalized by the vessel area. (**D**,**E**) Hydrogen peroxide production measured using Amplex Red probe in HAECs (**D**) shown as time-course of fluorescence and total area under the curve (*n* = 6, * *p* < 0.01) and in mouse aortas (**E**) shown as total production in 1 h (*n* = 4–5, * *p* < 0.05). (**F**) Protein from aortic lysates was extracted and used to determine superoxide dismutase (SOD2) levels by Western blotting analysis (*n* = 2 experiments, * *p* < 0.05). (**G**) Oxidized/reduced glutathione ratio in aorta, *n* = 4, * *p* < 0.05. Data are expressed as mean ± SEM. HAECs, human aortic endothelial cells. CETP, cholesteryl ester transfer protein. NS, non-silenced. siCETP, silenced CETP, FACs, fluorescence assisted cell sorted, TBHP, tert-Butyl hydroperoxide.

To determine the molecular underpinnings for impaired endothelial cell function under CETP, we examined ROS generation. CellRox staining followed by FACs, demonstrated reduced oxidative stress after CETP silencing on HAECs (Figure 3A). We also investigated the role of CETP separately on superoxide and hydrogen peroxide production using the DHE probe and the Amplex Red/horseradish peroxidase assay. Notably, after CETP silencing of HAECs, superoxide production was reduced by 80% (Figure 3B) and in-situ superoxide generation in aorta sections from CETP-transgenic mice was 50% higher than in Ntg mice (Figure 3C). Hydrogen peroxide levels both in vitro and in vivo, correlated with CETP expression. In HAECs, hydrogen peroxide was reduced by 23% after silencing CETP (Figure 3D), whereas aortas of CETP Tg mice have 45% more hydrogen peroxide compared to Ntg mice (Figure 3E). These results were further corroborated by the ~50% increase in endogenous antioxidant enzyme SOD2 protein levels in aortas from CETP-transgenic mice (Figure 3F). Glutathione represents one of the most abundant reducing agents for maintaining cell redox homeostasis. We measured the level of reduced glutathione in aortas from CETP-transgenic and non-transgenic mice. Our data showed that aortas from CETP-transgenic mice have an increased ratio of oxidized to reduced glutathione (GSSG/GSH), suggesting that CETP accelerates GSH oxidation in vivo (Figure 3G).

**Figure 4:**
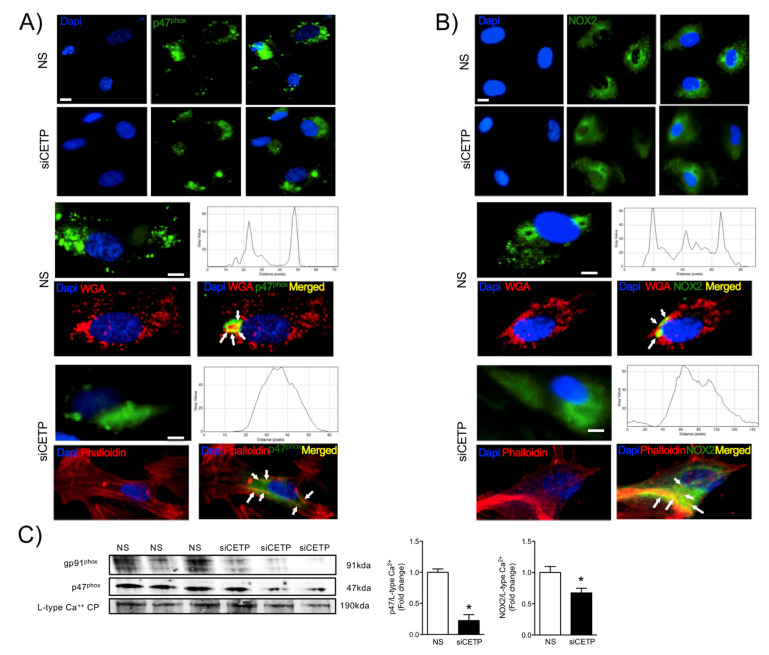
CETP inhibition decreases NADPH oxidase activation. Representative microscopic images of HAEC stained with (**A**) p47^phox^ (**B**) NOX2 (gp91^phox^) and DAPI. p47^phox^ and NOX2 (green) are localized to the plasma membrane, stained with wheat germ agglutinin (WGA-red). After CETP inhibition, p47^phox^ and NOX2 co-localize with cytoplasmic aggregates of endogenous actin, detectable with phalloidin labeling. Upper panel 10X magnification and bottom 20X. Scale bar, 10 μm. (**C**) Western blotting of p47^phox^ and NOX2 (gp91^phox^) in the plasma membrane fraction of HAEC normalized by the L-type calcium channel protein (*n* = 3, * *p* < 0.05). HAECs, human aortic endothelial cells. CETP, cholesteryl ester transfer protein. NS, non-silenced. siCETP, silenced CETP.

NADPH oxidases (NOX), a multi-unit enzymatic complex, is an important source of ROS in endothelial cells. Assembly of the active NOX complex requires the association of the cytosolic and plasma membrane subunits. The expression and co-association of these subunits are a useful index of NOX activation. Thus, we investigated the localization of p47^phox^ (cytoplasmic subunit) in response to the silencing of CETP. We verified that, in response to CETP silencing, p47^phox^ and NOX localizes mainly to the cytosol (Figure 4A) and co-localizes with cytoplasmic aggregates of actin, detectable with phalloidin labeling as compared to control HAEC fully expressing CETP (Figure 4A), where the distribution was in the cell membrane and co-localise with wheat germ agglutinin. By Western-blot of HAEC cell membranes, we confirmed that inhibition of CETP (siCETP) decreased the abundance of p47^phox^ in the cell membrane (Figure 4C), indicating less translocation of p47^phox^ to the membrane. In addition, NOX2 (gp91^phox^, membrane subunit) protein expression in HAEC cell membranes is also decreased by CETP inhibition (Figure 4B,C). Collectively, these results suggest that CETP leads to increased NOX2 activation and/or expression.

**Figure 5:**
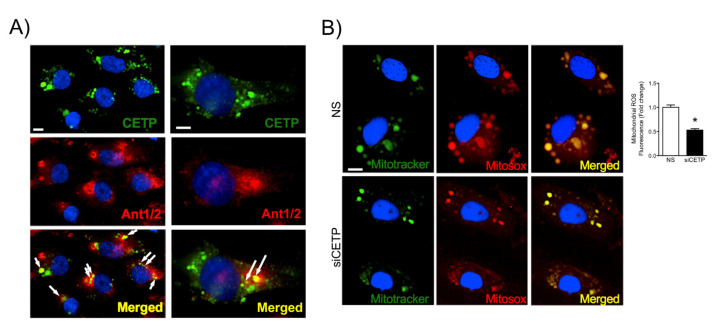
CETP co-localizes partially with mitochondria and increases mitochondrial superoxide production. (**A**) Representative images of HAECs showing CETP in green, mitochondrial carrier protein adenine nucleotide translocator (Ant1/2) in red, and DAPI in blue (nuclear dye). Arrows indicate co-localization of CETP and ANT1/2 (yellow). Magnification 10X left and 20X right. Scale bar, 10 μm. (**B**) Representative images of HAEC after CETP silencing and staining with MitoSOX Red (mitochondrial superoxide indicator), Mitotracker Green (mitochondrial marker), and DAPI. Magnification is 10X and x2 zoom. Scale bar, 10 μm. Quantification of co-localization of Mitotracker and Mitosox (*n* = 5, * *p* < 0.001). HAECs, human aortic endothelial cells. CETP, cholesteryl ester transfer protein. NS, non-silenced. siCETP, silenced CETP.

Mitochondria are major sites of cell ROS production and also for antioxidant defenses. We assessed the co-localization of CETP with mitochondria by labeling with the adenine nucleotide translocator (ANT), which is a mitochondrial carrier protein that exchanges ATP and ADP between the cytoplasm and the mitochondrial matrix. We observed that a considerable amount of CETP was co-localized with ANT1/2 in HAEC (Figure 5A). In line with these observations, we further examined the effect of silencing CETP on the net production of mitochondrial superoxide in HAECs using MitoTracker Green and MitoSox Red staining and observed that siRNA-mediated knockdown of CETP decreased mitochondrial superoxide production by 47% (Figure 5B).

### 3.4. CETP Inhibition Downregulates Endoplasmic Reticulum Stress

**Figure 6:**
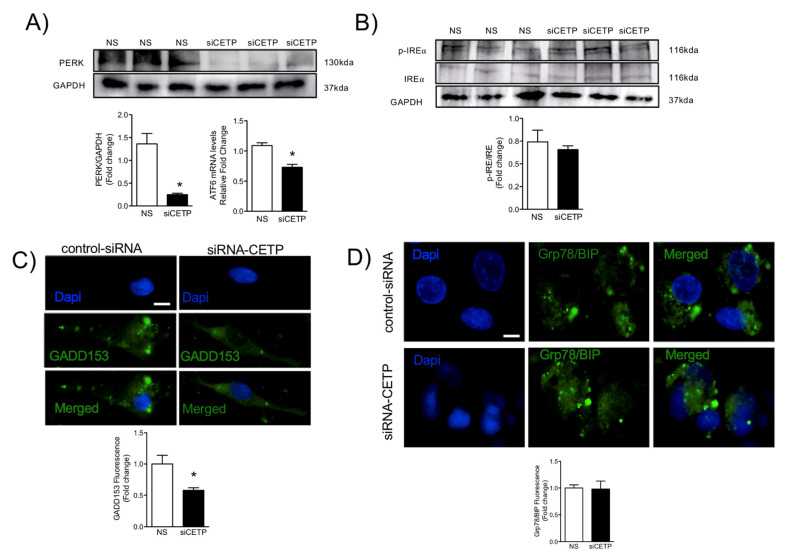
CETP inhibition reduces endoplasmic reticulum stress. HAECs transfected with CETP or control scrambled siRNA. (**A**) Western blot analysis of PERK (*n* = 3, * *p* < 0.01) and qPCR analysis of ATF6 mRNA. (**B**) Phosphorylated and total IRE (*n* = 3, ns). (**C**) Representative microscopic images of HAECs stained with GADD153, *n* = 5, and (**D**) Grp78/BIP, and DAPI. Scale bar, 10 μm. Mean ± SEM, *n* = 5, * *p* < 0.05. HAECs, human aortic endothelial cells. CETP, cholesteryl ester transfer protein. NS, non-silenced. siCETP, silenced CETP. PERK, protein kinase RNA-like ER kinase. ATF6, Activating transcription factor 6. IRE, inhibitor resistant esterase. BIP, binding immunoglobulin protein. GADD153, growth arrest- and DNA damage-inducible gene 153.

We further investigated whether CETP could elicit endoplasmic reticulum (ER) stress, by evaluating unfolded protein response (UPR)-markers. Analysis of gene and protein expression of multiple UPR markers in endothelial cells after CETP silencing revealed deactivation of UPR sensors and effectors. In endothelial cells, we detected reduced levels of the ARF6 transcript (30%) and a marked deactivation of the protein kinase RNA-like ER kinase (PERK) UPR branches (Figure 6A), but no changes in IRE1α phosphorylation (Figure 6B). Notably, reducing CETP promoted a significant reduction in the protein expression of CCAAT-enhancer-binding protein homologous protein (CHOP), also known as GADD153 (41%) (Figure 6C), but no changes in the protein expression of binding immunoglobulin protein (GRP78/BIP) (Figure 6D). These results implicate a role of CETP in the ER stress response, which can superimpose with other metabolic drivers of inflammation.

### 3.5. Removal of CETP Reduces Cell Adhesion Molecules in Endothelial Cells

**Figure 7:**
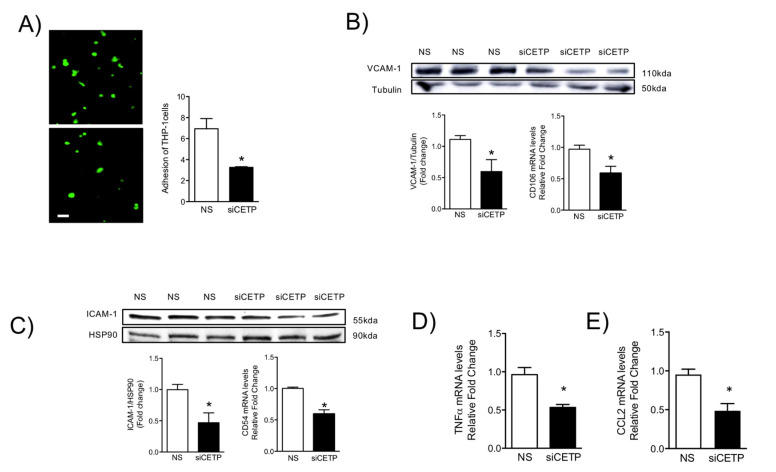
CETP inhibition reduces adhesion molecules expression and monocyte adhesion in endothelial cells. (**A**) Fluorescently stained THP-1 cells adhered to the monolayer of HAECs transfected with siCETP or control scrambled siRNA. The average number of THP-1 cells adhered on the monolayer of HAECs in a field with the same size was quantified and results are expressed as Mean ± SEM (*n* = 3), * *p* < 0.05. Protein expression and mRNA levels of adhesion molecules in HAECs: (**B**) VCAM-1 immunoblot and qPCR analysis of CD106 mRNA, (**C**) ICAM-1 immunoblot and qPCR analysis of CD54 mRNA. Protein levels were normalized to Tubulin or HSP90 (* *p* < 0.05). (**D**,**E**) qPCR analysis of TNFA and CCL2 mRNA in HAECs transfected with siCETP or control scrambled siRNA. Results are expressed as Mean ± SEM (*n* = 3), * *p* < 0.05. HAECs, human aortic endothelial cells. CETP, cholesteryl ester transfer protein. NS, non-silenced. siCETP, silenced CETP.

Since leukocyte attraction, adhesion and infiltration into the vessel wall may be a consequence of ROS-induced endothelial activation [19], we examined monocyte adhesion onto HAECs. Originally, endothelial activation was defined as the increased expression of endothelial adhesion molecules [20]. The activation, injury, and dysfunction of endothelial cells are a common trilogy for many cardiovascular diseases. We verified that the presence of CETP is associated with dysfunction and activation of endothelial cells. The monocyte adhesion assay was conducted in HAECs and the THP-1 monocyte co-culture and showed decreased monocyte trafficking onto HAECs following CETP knockdown (Figure 7A). This effect was correlated with reduced gene and protein expression of the cell adhesion molecules, VCAM-1 (Figure 7B) and ICAM-1 (Figure 7C). Silencing CETP significantly down-regulated the gene (CD106) and protein expression of VCAM-1 (Figure 7B, 60%) and gene (CD54) and protein expression of ICAM-1 (Figure 7C, 53%) in HAECs. We further investigated inflammatory cytokine TNFα and CCL2 chemokine gene expression, which encodes monocyte chemoattractant protein 1 (MCP1) (Figure 7E). The basal TNFα and CCL2 mRNA levels in HAECs were reduced by 50% and 48% in siCETP cells, respectively (Figure 7D). These findings indicate that CETP plays a role in the recruitment of leukocytes.

## 4. Discussion

Endothelial dysfunction, which is the earliest detectable change in the initiation of atherosclerosis [21], is a central feature of many CVDs and is used as an independent predictor of morbidity/mortality in many clinical studies [22,23,24]. The purpose of the present study was to investigate whether and to what extent CETP expression impacts endothelial cell function, which is a well-established surrogate marker for CVD risk. Using a mouse model that recapitulates the myriad features of human endothelial cells expressing CETP [25,26], we uncovered evidence that CETP expression is associated with endothelial dysfunction, and enhances ROS production, eNOS phosphorylation, endoplasmic reticulum stress, and expression of cell adhesion molecules.

The reduced maximum relaxation in response to acetylcholine in aortas from CETP-transgenic mice, support that this mouse represents a model of endothelial dysfunction. In addition, L-NAME incubation abolished the endothelium-dependent relaxation in response to acetylcholine in both groups, highlighting the importance of NOS. Intriguingly, CETP transgenic aortas also expressed increased eNOS phosphorylation on Ser1177 and reduced eNOS/Cav1 and nNOS/Cav3 interaction, suggesting activation of the constitutive NOS isoforms. Similar findings were seen in the experiments using HAECs, where CETP expressing cells showed higher eNOS phosphorylation compared to cells after CETP knockdown. These findings agree with the previous studies from Gomaraschi and colleagues where HDL from CETP-deficient subjects had a reduced capacity to activate eNOS due to lower bioactive components PON-1 (enzyme that plays a role in eNOS activation) and S1P (lysosphingolipid) [27,28]. Our data also showed a decrease in lipid rafts content in HAECs associated with CETP inhibition and this finding could be linked with the regulation of expression or activity of eNOS in cell membranes. We also speculate that higher eNOS phosphorylation and activation in the presence of CETP could be dysfunctional. Increased superoxide generation inactivates NO, reducing its bioavailability and increasing peroxynitrite (ONOO^-^) [29,30]. The activation of eNOS under oxidative stress state can cause enzyme uncoupling and a shift to superoxide production with less NO synthesis [31,32]. eNOS form a functional dimer to couple haem and O_2_ reduction to synthetize NO, but oxidative stress may impact eNOS dimerization and coupling [33]. Therefore, oxidative stress might mitigate the CETP stimulation of NOS activity. A limitation of this study includes the absence of eNOS dimerization/coupling analysis.

The findings from this work in vitro and in vivo demonstrate that endothelial cells expressing CETP have increased superoxide and hydrogen peroxide production rates and that GSH oxidation is accelerated. To distinguish the possible sources of ROS, we investigated the state of activity of NADPH oxidase (NOX), which is one of the major sources of ROS in vascular cells. Although several isoforms of NOX are expressed in the vessel endothelium, accumulating evidence suggests a crucial role of NOX2 in endothelial injury [34]. NOX2 is the gp91^phox^ membrane subunit analog that assembles membrane p22^phox^ and cytosolic p47^phox^, p40^phox^, p67^phox^, and Rac1 subunits. In response to the cellular stress, the cytosolic p47^phox^ translocates to the plasma membrane and binds to the membrane-bound catalytic domain, activating NOX2 [35]. Our analyses suggest that, when CETP is inhibited, there is less NOX2 expression, less translocation of the cytoplasmic subunit p47^phox^ to the plasma membrane, and, thus, less NOX2 activation. There is a crosstalk between activated NOX and mitochondrial ROS production [36], and our data showed markedly increased mitochondrial superoxide production in CETP expressing cells. Therefore, we presume the existence of a vicious cycle of ROS generation in these cells. Experiments using NOX inhibitors (Apocynin or DPI) or antioxidant mitoTempo abolished CETP dependent differences in ROS production (data not shown).

ER stress is another feature of dysfunctional endothelium [37]. The unfolded protein response (UPR) is an evolutionary conserved adaptive response capable of mitigating stress in conditions that disrupt protein quality control. IRE1α is the most evolutionary conserved branch of UPR, which is one that has recently been identified as a regulator of stress responsiveness. In the present study, UPR sensors and effectors were deactivated after the silencing of CETP, as shown by the reduction in activating transcription factor 6 (ATF6) and the deactivation of the protein kinase RNA-like ER kinase (PERK) UPR branch. One of the components of the ER stress-mediated apoptosis pathway is a C/EBP homologous protein (CHOP), also known as growth arrest- and DNA damage-inducible gene 153 (GADD153) [38], which was reduced after silencing of CETP, indicating that CETP expression is involved in ER stress, likely through elevation of ROS production. The potential role of CETP in modulating ER stress is particularly intriguing, and our results are in accordance with Lira et al. 2008 [39], who concluded that CETP transfection in the human liposarcoma cell line (SW872) and human embryonic kidney cell line (HEK293S) causes an induction of genes linked to the ER stress response.

Oxidative stress and inflammation are tightly associated with endothelial activation [40]. One feature of an activated endothelium is the upregulation of adhesion molecule expression [41]. These adhesion molecules promote the recruitment of circulating monocytes that transmigrate into the sub-endothelial space, differentiate into macrophages, and become foam cells under atherogenic conditions. We showed that CETP inhibition in HAECs resulted in reduction of TNFα, CCL2, adhesion molecule ICAM-1, and VCAM-1 expression and monocyte trafficking. Together, these results suggest that CETP expression leads to EC dysfunction and may play a role in the setting of EC activation, which is a process that promotes the expression of adhesion molecules, resulting in the recruitment of monocytes/macrophages that could accelerate atherosclerosis.

The CETP effects on endothelial cells described here seem to be independent of HDL, since most of them were observed in in vitro and ex vivo systems. Thus, we hypothesize that CETP has a local intracellular function. Together with our results, the study of Lira and collaborators (2008) [39] showing CETP induces ER stress support this proposition. In addition, Izem and Morton (2007) [42] have shown that CETP expression modulates cholesterol and triglyceride homeostasis in the SW872 human liposarcoma cell line. They showed that CETP-deficient cells had inefficient CETP-mediated translocation of cholesterol and triglycerides from the ER to their site of storage. Thus, it is possible that CETP, as a lipid transfer protein, may affect the lipid composition and/or the amount of specific membrane domains highly relevant for endothelial cells. Our data show that inhibition of CETP decreases significantly lipid raft markers in HAECs, which may explain the effects on caveolin/NOS association and the eNOS activation state.

Taken together, our data suggest that CETP-driven endothelial cell oxidative stress is promoted by the major endothelial cell sources of ROS: NOX2 and mitochondria. We propose that CETP-induced ROS production leads to a reduced NO bioavailability and impaired endothelial function even in the presence of nNOS and eNOS activation. CETP induced ER stress is also associated with endothelial dysfunction. Considering that global CETP inhibition has not reached the expected benefits [43,44], the present results suggest that endothelial CETP inhibition may be a good strategy to decrease atherosclerosis risk.
